# Upregulated ATG4B predicts poor prognosis and correlates with angiogenesis in osteosarcoma

**DOI:** 10.1186/s43046-025-00269-z

**Published:** 2025-04-25

**Authors:** Elzahraa Ibrahim Mohamed Khalil, Fatma El Zahraa Ammar Mohamed, Rehab Kamal Mohamed

**Affiliations:** 1 Pathology Department, Faculty of Medicine, Minia University, Minia, Egypt; 2 Pathological Sciences Department, MBBS Program,, Fakeeh College for Medical Sciences, Jeddah, Saudi Arabia; 3 Pathology Department, Faculty of Medicine, Minia University, Minia, Egypt

**Keywords:** ATG4B, VEGF, Autophagy, Angiogenesis, Osteosarcoma

## Abstract

**Background:**

Osteosarcoma (OS) is the most common primary bone cancer in children and adolescents. Between 35 and 45% of these patients do not respond to standard chemotherapeutic treatments, resulting in a very low 5-year survival rate of only 5–20%. This resistance often leads to treatment failure and unfavorable prognoses, highlighting the critical need for new therapeutic targets to improve treatment strategies. Autophagy-related gene 4 B (ATG4B) is a crucial cysteine protease for autophagosome formation. It is overexpressed and correlates with poor prognosis in various cancers. However, the relationship between ATG4B expression and angiogenesis in OS remains unexplored. This study investigated the expression levels of ATG4B and VEGF in OS and their correlation with clinicopathological data.

**Materials and methods:**

This study included 67 paraffin-embedded OS tissue samples. ATG4B and VEGF expression levels were assessed via immunohistochemistry, and their associations with clinicopathological variables were statistically analyzed. Additionally, ATG4B gene expression in OS was examined via GEO datasets from https://www.ncbi.nlm.nih.gov.

**Results:**

ATG4B and VEGF were expressed in 79.1% and 74.6% of the osteosarcoma samples, respectively. There was a significant positive correlation between ATG4B expression and tumor size, tumor stage, and histological response to neoadjuvant chemotherapy, with *p* values of 0.013, 0.008, and 0.022, respectively. VEGF expression was also significantly correlated with tumor size, tumor stage, and the presence of distant metastasis at diagnosis, with *p* values of 0.022, 0.044, and 0.013, respectively. A notable positive correlation between ATG4B and VEGF expression levels was observed (*p*=0.002), which was supported by the GEO dataset analysis. High ATG4B and VEGF overexpression were significantly associated with worse overall survival by univariate analysis.

**Conclusions:**

The results suggest that ATG4B acts as a tumor promoter in OS, indicating its potential as a therapeutic target to inhibit tumor growth. Elevated ATG4B levels may also serve as a marker for poor prognosis. Additionally, VEGF overexpression is linked to a greater likelihood of pulmonary metastasis and a worse overall prognosis. The positive correlation between ATG4B and VEGF suggests that the absence of both markers could be indicative of a better chemotherapy response, offering insights into potential new treatment approaches.

## Introduction

Osteosarcoma (OS) represents the predominant primary bone malignancy in children and adolescents, constituting 3–4% of all malignancies [1] and approximately 15% of all bone malignancies [2]. It is a disastrous tumor characterized by brisk growth, local aggressiveness, and early pulmonary metastasis [3]. Currently, patients with metastases face significantly poorer outcomes, largely attributed to chemotherapy drug resistance [4, 5]. A substantial portion of OS patients, ranging from 35–45%, do not respond to chemotherapeutic drugs, resulting in a mere 5–20% 5-year survival rate. Chemoresistance frequently leads to treatment failure and unfavorable prognoses [6, 7]. Consequently, there is a pressing need to explore novel therapeutic targets for the development of innovative treatment strategies for OS.

Autophagy, a conserved process that facilitates the degradation and recycling of altered cellular components and organelles, has been demonstrated to promote cell survival or death, underscoring its critical role in tumor development [8, 9]. The role of autophagy in various cancers remains a subject of debate; its impact on cancer is multifaceted and contingent upon the tumor stage and type. While autophagy can function as a tumor suppressor, it may also act as a tumor promoter by enhancing the survival of cancer cells under conditions of nutrient deprivation stress [10, 11]. Moreover, numerous researchers have proven that autophagy-mediated chemotherapy resistance occurs during cancer treatment [12, 13].

Autophagy-related gene 4 (ATG4) serves as a cysteine protease crucial for autophagosome formation and comprises four homologs—ATG4A, ATG4B, ATG4C, and ATG4D. In particular, ATG4B functions as an essential protease, facilitating the cleavage of pro LC3 or lipidated LC3-II for proper autophagosome formation [14]. Previous investigations have revealed that ATG4B is overexpressed and correlates with poor prognosis in chronic myeloid leukemia, breast cancer, gastric cancer and oral cancer [15–18]. Additionally, an increasing number of studies suggest an oncogenic role for ATG4B in colorectal carcinoma and glioblastoma [19, 20]. Inhibition of ATG4B activity has been shown to reduce cancer cell viability and increase the sensitivity of cancer cells to chemotherapeutic agents both in vitro and in vivo [21–23].

A few studies have explored the role of ATG4B in OS. Interestingly, OS Saos-2 cells lacking ATG4B exhibited defects in autophagy and failed to develop tumors in mouse models. Moreover, the resistance of OS cell lines to doxorubicin, cisplatin, and methotrexate has been attributed to the induction of autophagy [23]. Given the potential of autophagy-based strategies in targeted tumor therapies and the importance of understanding the clinical relevance of ATG4B in OS patients, we conducted a comparative analysis of ATG4B protein levels via immunohistochemistry alongside clinical outcomes in OS patients.

Angiogenesis, a hallmark of tumors, plays a vital role in facilitating sustained tumor growth and dissemination [24]. This process is tightly regulated by a delicate balance between proangiogenic and antiangiogenic factors. Among these factors, vascular endothelial growth factor (VEGF), a homodimeric protein, acts as a specific mitogen for endothelial cells and is often found to be overexpressed in various inflammatory diseases and tumors. Notably, the tumor microenvironment (TME) is largely influenced by the VEGF-VEGFR2 axis [25, 26]. Understanding the regulation and influencing factors of the TME in OS is essential for developing more targeted therapeutic strategies and improving the prognosis and quality of life of patients [27].

Emerging evidence highlights the crucial role of autophagy in endothelial cells (ECs). Moreover, genetic findings in recent years indicate that autophagy regulates pathological angiogenesis, a hallmark of solid tumors. In the hypoxic, nutrient-deprived, and proangiogenic TME, increased autophagy in blood vessels is emerging as a critical mechanism enabling endothelial cells to evade hypoxia-induced cell death [26]. However, the relationship between ATG4B expression and angiogenesis in tumor cells has still not been elucidated. Therefore, via immunohistochemistry and gene microarray analysis, this study aimed to investigate the expression levels of ATG4B and VEGF in OS and their relationships with clinicopathological data. Additionally, the correlations among the immunohistochemical markers were examined.

## Materials and methods

### Patients

This study included a total of 67 primary OS patients, who were randomly selected. Those patients were sourced from the Pathology Department of Minia University Hospital and Minia Oncology Center in Egypt, spanning from 2016–2022. Clinical data and follow-up information were retrieved from the patients’ medical records with the approval of the respective hospital districts. Furthermore, the study protocol received approval from the Ethics Committee of Minia University, Faculty of Medicine, Institutional Review Board (MUFMIRB 1204/07/2024). Patients included in the study underwent both chemotherapy and surgical treatment as part of their management regimen.

### Histopathological evaluation

Representative hematoxylin and eosin (H&E)-stained slides from all patients were meticulously reviewed to validate the original diagnosis. Staging was performed following the Enneking staging system for malignant musculoskeletal tumors [28]. Additionally, the response to preoperative neoadjuvant chemotherapy was histologically evaluated on the basis of the Huvos grading system [29]. Patients received neoadjuvant chemotherapy via the SEOP-95 regimen, which comprises high-dose methotrexate, adriamycin, cisplatin, and ifosfamide. Overall survival was calculated in months from the date of diagnosis to the time of tumor-related death or the last follow-up visit.

### Expression of ATG4B in a publicly available dataset. (Dataset analysis)

The expression of ATG4B was investigated in OS samples from the Gene Expression Omnibus (GEO2R) dataset at https://www.ncbi.nlm.nih.gov. Gene expression values were extracted from every dataset by comparing two groups, i.e., OS and adjacent nontumor tissues, and the expression value of the gene in the microarray was subsequently calculated via GEO2R. The following datasets were investigated: GSE126209 and GSE56001. The analysis was performed as described in previous research [30].

### Immunohistochemistry

Immunohistochemistry was conducted on 4-µm tissue sections mounted on positively charged slides. The sections were deparaffinized and rehydrated through xylene and graded ethanol solutions. To block endogenous peroxidase activity, the sections were treated with hydrogen peroxide for 20 min. Antigen retrieval was performed by treating the sections with 0.1 mol/L citrate buffer (pH 6.0) in a 700 W microwave oven for 20 min. The sections were subsequently incubated overnight at 4 °C with the following primary antibodies: ATG4B (dilution 1:100; A2981, Sigma‒Aldrich, St. Louis, MO, USA) and the monoclonal mouse VEGF antibody Ab-7 (clone VG1, ready to use; Thermo Fisher Scientific/Lab Vision Corporation, USA). The reaction was visualized via an avidin–biotin detection kit with diaminobenzidine (DAB) as the chromogen. Finally, the sections were counterstained with Mayer’s hematoxylin.

For negative controls, nonspecific activity was evaluated by omitting the primary antibodies and incubating the slides with phosphate-buffered saline (PBS). Human normal kidney tissue served as a positive control for the ATG4B antibody, whereas endothelial cells of capillaries within the stained sections served as an internal positive control for the VEGF antibody.

### Immunohistochemical scoring

All slides were reviewed and independently scored by two pathologists. The ATG4B immunoreactivity of the tumor cells was measured via semiquantitative estimation; a score was obtained by adding the values of the percentage and intensity of stained tumor cells. The intensity was graded as 0, negative; 1, weak; 2, moderate; and 3, intense. The percentage of positive cells was graded from 0 to 3 (0: nil; 1: 1–25%; 2: 26–50%; 3: > 51%). Low ATG4B expression was defined if the score was between 0 and 4, whereas cases with scores of 5 or 6 were considered high ATG4B expression [31]. VEGF immunoreactivity was scored on the basis of the percentage of positive tumor cells: high VEGF expression (> 30% of tumor cells) and low VEGF expression (≤ 30% of tumor cells) [30].

### Statistical analysis

Chi-square tests and Fisher’s exact tests were employed to detect differences in categorical variables. The results were considered statistically significant when the *p* value was < 0.05. Patient survival and differences were assessed via the Kaplan–Meier method. A multivariate Cox proportional hazards regression analysis was conducted to evaluate the specific impact of each variable on survival in the presence of other variables. Data analysis was performed via the Statistical Package for Social Sciences (SPSS) version 21 software.

## Results

### Expression of ATG4B in a publicly available dataset. (Dataset analysis)

**Fig. 1:**
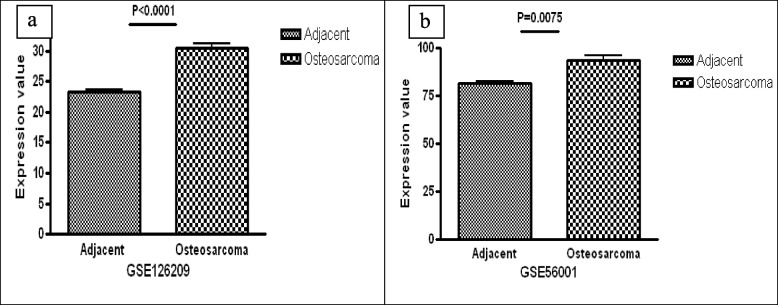
Gene Expression Omnibus database (GEO2R) data sets analysis for ATG4B expression in osteosarcoma tissue and normal human osteoblast: ATG4B expression significantly increased in OS tissue compared to normal osteoblast in GSE126209 (**a**) and GSE56001 (**b**) data sets

A comparison of the expression of ATG4B in OS tissue and surrounding nontumor tissue revealed a significant increase in ATG4B gene expression in OS tissue in two datasets, GSE126209 and GSE56001 (*P* < 0.0001 and *P* = 0.0075, respectively) (Fig. 1).

### Patient and tumor characteristics

In the present study, the mean age of patients was 14 years, with a standard deviation of 1.9 years. The median age was 12 years, with a range of 9–18 years. Tumor size was categorized into two groups, ≤ 9 cm and > 9 cm, on the basis of the median value. Among the patients, 56.7% (38 out of 67) received neoadjuvant chemotherapy. Among these patients, 13 were classified as poor responders (necrosis less than 90%), whereas 25 were classified as good responders (necrosis equal to or more than 90%). The detailed characteristics of the patients are provided in Table 1.

**Table 1:** Clinicopathological features of OS patients

Clinicopathological Features (*N* = 67)	Number %
Age at diagnosis	
≥ 14 years	37 (55.2)
> 14 years	30 (44.8)
Gender	
Male	43 (64.2)
Female	24 (35.8)
Location of tumor	
Extremities	61(91)
Axial	6 (9)
Tumor Size	
≤ 9cm	43 (64.2)
> 9cm	24 (35.8)
Histological subtype	
Osteoblastic	37 (55.2)
Chondroblastic	26 (38.8)
Fibroblastic	6 (6)
Stage	
I	4 (6)
II	50 (74.6)
III	13 (19.4)
Distant Metastases at the onset of diagnosis	
No	54 (80.6)
Yes	13 (19.4)
Histological response to chemotherapy (*n*. = 38)	
≥ 90%	21(55.2)
< 90%	17 (44.8)

### Immunohistochemical results of ATG4B and VEGF

**Fig. 2:**
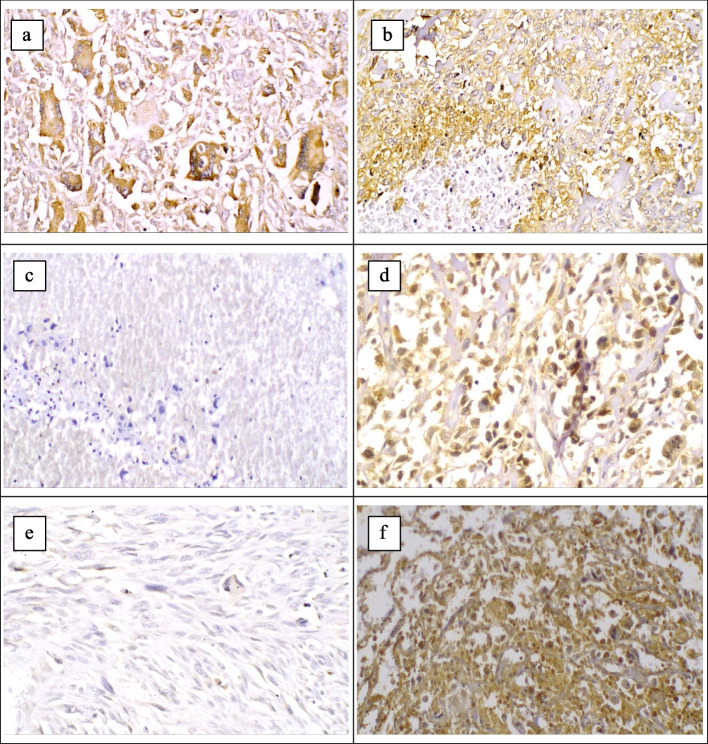
Immunohistochemical expression of ATG4B and VEGF in primary and metastatic osteosarcoma. **a** High expression of ATG4B in the cytoplasm of OS cells. **b** High expression of ATG4B in a case with poor response to neoadjuvant chemotherapy. **c** Low expression of ATG4B in a case with good response to neoadjuvant chemotherapy. **d** Positive expression of VEGF in primary osteosarcoma. **e** Negative expression of VEGF in primary osteosarcoma. **f** Positive expression of VEGF in metastatic osteosarcoma (**f**). Immunohistochemistry: Diaminobenzidine (DAB) chromogen and Mayer’s hematoxylin counterstaining. Original magnifications are 400x·

ATG4B protein expression was detected in the cytoplasm of tumor cells. Among the 67 OS patients tested, 53 (79.1%) exhibited high ATG4B immunoreactivity. Significant positive relationships were identified between high ATG4B expression and tumor size, stage, and poor histological response to neoadjuvant chemotherapy (with *p* values of 0.013, 0.008, and 0.022, respectively) (Fig. 2a-c). No significant relationships were identified between the expression of ATG4B and patient age, sex, or histological subtype. Moreover, no significant difference in ATG4B expression was noted based on the location of the tumor or the presence of distant metastasis at the time of diagnosis. However, a higher incidence of high ATG4B expression was observed in axial OS than in extremity OS (100% versus 77%) and in metastatic OS than in nonmetastatic OS (92.3% versus 75.9%).

Positive cytoplasmic VEGF immunoreactivity was observed in 50 out of 67 tumors (74.6%). High VEGF expression was significantly associated with tumor size, tumor stage and the presence of distant metastasis at the time of diagnosis (*p *values of 0.022, 0.044 and 0.013, respectively) (Fig. 2d-f). There was no significant association between VEGF expression and other clinicopathological features.

The relationships of ATG4B and VEGF expression with different clinicopathological features are summarized in Table 2.

**Table 2:** Associations of ATG4B and VEGF with clinicopathological data

Clinicopathalogical characteristics	N	ATG4B		*P*value	VEGF		*P* value
		High	Low		High	Low	
Age at diagnosis							
≥ 14 years	37	28 (75.6%)	9 (24.4%)	0.4	29 (56.7%)	16 (43.3%)	0.67
> 14 years	30	25 (83.4%)	5 (16.6%)		21 (70%)	9 (30%)	
Gender							
Male	43	32 (74.4%)	11 (25.6%)	0.2	30 (69.3%)	13 (30.7%)	0.22
Female	24	21 (87.5%)	3 (12.5%)		20 (83.3%)	4 (16.7%)	
Location of tumor							
Extremities	61	47 (77%)	14(23%)	0.18	44 (72.1%)	17 (27.9%)	0.13
Axial	6	6 (100%)	0		6 (100%)	0	
Tumor Size							
≤ 9 cm	43	38 (88.4%)	5 (11.6%)	**0.01**	36 (83.7%)	7 (16.3%)	**0.02**
> 9 cm	24	15 (62.5%)	9 (37.5%)		14 (58.3%)	10 (41.7%)	
Histological subtype							
Osteoblastic	37	29 (78.3%)	8 (21.7%)	0.48	27 (72.9%)	10 (27.1%)	0.41
Chondroblastic	26	20 (76.9%)	7 (23.1%)		18 (69.2%)	8 (30.8%)	
Fibroblastic	6	4 (66.7%)	2 (33.3%)		5 (83.3%)	1 (16.7%)	
Stage							
I	4	1 (25%)	3 (75%)	**0.01**	2 (50%)	2 (50%)	**0.04**
II	50	40 (80%)	10 (20%)		35(70%)	13 (30%)	
III	13	12 (92.3%)	1 (7.7%)		13 (100%)	0	
Distant Metastases at the onset of diagnosis							
No	54	41 (75.9%)	13 (24.1%)	0.19	38 (70.4%)	14 (29.6%)	**0.01**
Yes	13	12 (92.3%)	1 (6.7%)		12 (92.3%)	1 (6.7%)	
Histological response to chemotherapy (n. = 38)							
≥ 90%	21	15 (71.4%)	6 (28.6%)	**0.04**	14 (66.7%)	7 (33.3%)	0.5
< 90%	17	16 (94.1%)	1 (5.9%)		13 (76.5%)	4 (23.5%)	

Test of significance: Chi-square test and Fisher’s exact test, *P*-value < 0.05 is considered significant

### Relationship between ATG4B and VEGF

A statistically significant positive correlation was found between ATG4B and VEGF expression (*P* = 0.002), where a significant proportion of high VEGF cases (65.6%) had high ATG4B expression scores.

Moreover, the combined expression patterns of both ATG4B and VEGF with respect to the histological response to chemotherapy in each tumor were studied, and we found a significant association between a low ATG4B/low VEGF expression pattern and a good histological response to chemotherapy (*P* = 0.02), where all tumors with low expression of both markers were associated with a good histological response to chemotherapy. (Table 3).

**Table 3:** Combined ATG4B and VEGF expression in association with histological response to chemotherapy

	** ≥ 90% necrosis**	** < 90% necrosis**	***P* value**
High ATG4B/ High VEGF	12 (50%)	12 (50%)	**0.025**
Low ATG4B/ Low VEGF	4 (100%)	0	
High ATG4B/ Low VEGF or Low ATG4B/ High VEGF	9 (90%)	1 (10%)	

Test of significance: Chi-square test, *P*-value < 0.05 is considered significant

### Follow-up and survival analysis

After a median follow-up of 5 years (range, 10–60 months), 4 patients (6%) were lost to follow-up during the study period. At the initial presentation, metastasis was detected in 13 patients (19.4%), whereas 21 patients (31.3%) experienced recurrent disease (3 local recurrences and 18 metastatic recurrences) during the follow-up period. According to the data cutoff, 26 patients (38.8%) died.

**Fig. 3:**
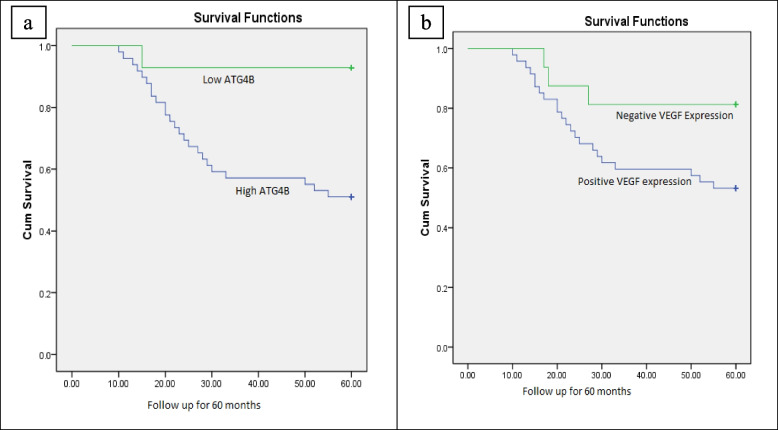
Kaplan Meier analysis of overall survival in osteosarcoma patients according to ATG4B (**a**) and VEGF (**b**) expression in osteosarcoma tissue samples

The 5-year overall survival rates were 68.6% and 16.7% for localized and metastatic OS patients, respectively. The Kaplan‒Meier method revealed significant associations between shorter overall survival in OS patients and high ATG4B expression (*p* = 0.01) (Fig. 3a), high VEGF expression (*p* = 0.049) (Fig. 3b), larger tumor size (*p* = 0.034), advanced stage (*P* < 0.001), the presence of metastasis at the time of diagnosis (*P* < 0.001), and poor histological response to chemotherapy (*P* < 0.001).

Multivariate Cox analysis of all predictors with *p* values < 0.05 in univariate analyses indicated that metastasis at the time of diagnosis, poor response to chemotherapy, and advanced stage were significant independent predictors of survival (see Table 4).

**Table 4:** Summary of univariate and multivariate analysis for overall survival

Predictor factors	Univariate	(95% CI)	Multivariate	(95% CI)
	***P***values		***P***values	
Age at diagnosis	0.62	(39.058- 48.402)		
14 years ≥				
> 14 years				
Gender	0.41	(41.058–50.502)		
Male				
Female				
Location of tumor	0.48	(44.050–47.802)		
Extremities				
Axial				
Tumor Size	**0.03**	(36.919–48.605)	0.39	(0.309–19.935)
≤ 9cm				
> 9cm				
Histological subtype	0.72	(36.058–42.502)		
Osteoblastic				
Chondroblastic				
Fibroblastic				
Stage	** < 0.001**	(16.058–34.324)	**0.03**	(0.035–0.845)
I				
II				
III				
Distant Metastases at the onset of diagnosis	** < 0.001**	(40.170- 52.101)	**0.03**	(0.035–0.845)
No				
Yes				
Histological response to chemotherapy (n. = 38)	** < 0.001**	(41.085- 50.502)	**0.02**	(0.051–0.785)
≥ 90%				
< 90%				
High ATG4B expression	**0.01**	(37.161–48.605)	0.93	(0.000–1.335)
High VEGF expression	**0.04**	(37.919–49.605)	0.95	(0.000–6.592)

*CI* confidence interval. Significant variables (*P* values < 0.05) are in bold

## Discussion

Despite significant improvements made over the past few decades in OS therapy, the 5-year survival rate of patients remains low, typically ranging from 15–20% [32]. Furthermore, patients with metastasis have an even worse prognosis. Therefore, understanding the mechanisms that drive the progression of OS presents new opportunities for improving patient prognosis and identifying novel therapeutic targets.

As mentioned earlier, autophagy may act as both a tumor suppressor and a tumor promoter [10, 11]. Among the ATG4 family, ATG4B serves as a functional protease that promotes autophagy activity [15]. Currently, there is limited knowledge about the role of ATG4B in the clinical outcomes of patients with OS. This study is the first to investigate the effect of the immunohistochemical expression of ATG4B on OS and its relationship with clinicopathological parameters. Additionally, this study aimed to explore the correlation between ATG4B and VEGF expression in OS tumor cells, shedding light on their potential interactions and implications for disease progression and treatment.

The role of autophagy in OS remains unclear; a dual role has been suggested [33]. Downregulation of autophagy-related genes such as ATG7 has been shown to inhibit cell proliferation in OS [34]. In contrast, Parlayan et al. [35] suggested that the activation of autophagy could inhibit the proliferation of OS cells by regulating BECN1 expression. These contrasting findings highlight the complexity of the involvement of autophagy in OS.

In the present study, ATG4B was significantly upregulated in OS osteoblasts compared with normal osteoblasts in the GSE12865 dataset. Additionally, our results align with studies conducted on OS cell lines, which demonstrated that OS Saos-2 cells lacking ATG4B failed to form tumors in mouse models. Moreover, researchers have developed an ATG4B antagonist that effectively suppresses tumor growth and induces tumor regression in OS xenografts [23]. These findings suggest that ATG4B may act as a tumor promoter in OS and that targeting ATG4B to inhibit autophagy could be a promising strategy for inhibiting tumor growth in OS.

Using immunohistochemistry, our findings revealed a significant positive association between ATG4B overexpression and adverse prognostic factors for OS, including increased tumor size and advanced disease stage. The present work also, found statistically.

significant relationship between high ATG4B expression and unfavorable overall survival by univariate analysis. These results are consistent with several studies across various tumor types, such as colorectal cancer, breast cancer, gastric carcinoma, chronic myeloid leukemia, glioblastoma and pancreatic carcinoma, which have demonstrated that higher ATG4B levels are indicative of poor prognosis and lower survival rates [16–20, 36]. These data strongly support the hypothesis that ATG4B plays a pivotal role in tumor growth and progression, making it a potential target for therapeutic intervention in various cancers, including OS.

Interestingly, we observed a significant positive association between high ATG4B expression and chemoresistance in OS. In recent years, the relationship between autophagy and the chemoresistance of tumor cells has gained significant attention. Our findings suggest that ATG4B may play a role in the development of chemoresistance and that its overexpression could serve as a predictor of poor response to neoadjuvant chemotherapy in terms of OS. It has been suggested that autophagy mediates chemoresistance to conventional anti-OS agents through high-mobility group box 1 protein (HMGB1) [37]. HMGB1 is overexpressed in OS tissue [38]. Notably, recent studies by Tan et al. [39] and Pu et al. [40] have demonstrated that the induction of protective autophagy by chemotherapy enhances the chemotherapeutic resistance of tumor cells. Moreover, a recent study revealed that the altered expression of various ncRNAs in OS cells can impact the response to chemotherapy by regulating processes such as cell apoptosis, signaling pathways, intracellular drug levels, and cell autophagy [41]. Therefore, autophagy may have a protective effect on OS cells against chemotherapeutic agents. Overall, our results provide further insight into the potential role of ATG4B in OS chemoresistance and highlight the complex interplay between autophagy and the chemotherapy response in this disease.

VEGF is well recognized for its role in metastasis, leading to substantial investigations into its expression in OS. Consistent with our findings, these studies reported that OS cells overexpress VEGF. Additionally, we found that higher levels of VEGF were significantly associated with larger tumor size, advanced stage and the presence of distant metastasis at the time of diagnosis. Moreover, we demonstrated statistically significant relationship between high VEGF expression and shorter overall survival by univariate analysis. These findings are supported by several reports indicating that VEGF overexpression is indicative of poor prognosis and lower survival rates, further supporting the hypothesis that VEGF is crucial for tumor growth and progression [30, 42–44].

In this study, a significant proportion of patients with high ATG4B expression exhibited VEGF overexpression. To better understand how pathological angiogenesis heightens autophagic flux, in solid tumors, including OS, angiogenesis is induced to supply the essential nutrients required for tumor cell energy demands and growth. The anoxic TME stabilizes hypoxia-inducible factors (HIFs), which regulate VEGF, inducing angiogenesis [45]. The imbalance between pro- and antiangiogenic signaling in the TME, which is primarily mediated by the VEGF/VEGFR2 axis, fuels pathological angiogenesis [46]. This results in unfavorable TME conditions characterized by an abundance of VEGF, nutrient deprivation, and aberrant blood flow, which consequently drive uncontrolled vessel sprouting and affect vessel maturation and function. As a consequence, nutrient deprivation, hypoxia, inflammation, and acidity occur within the TME. ECs embedded in the TME experience stressful conditions, leading to increased autophagic flux [47]. This heightened autophagy is a cellular response to cope with metabolic stress and promote cell survival [48, 49]. Moreover, autophagy has emerging functions in the secretion of cytokines [50] and angiogenesis-related factors [51]. Given these considerations, the positive correlation between high ATG4B and positive VEGF in OS tumor cells is not surprising. ATG4B, a key regulator of autophagy, may be upregulated in response to the stressful conditions, contributing to the survival and aggressive behavior of OS cells in the TME.

Regardless of whether pathological angiogenesis induces autophagy or vice versa, the abnormal vascular structure within the TME of OS significantly impedes the delivery of anticancer drugs, resulting in an uneven distribution of chemotherapeutic agents throughout the tumor [52], consequently limiting their therapeutic efficacy [53]. Clinical trials investigating targeted antiangiogenic drugs in OS patients have demonstrated that those with a low vascularization phenotype exhibit higher overall and relapse-free survival rates. Furthermore, patients with a low vascularization phenotype show a better response to neoadjuvant chemotherapy [54, 55].

An alternative perspective sheds light on the newly revealed role of extracellular vesicles (EVs) in the TME of bone sarcomas. These recent studies suggest that the microenvironment of OS cells transmits information through exosomes secreted by the cells. Specifically, the exosomal long noncoding RNA OIP5-AS1 has been identified as a key regulator of OS-associated angiogenesis and autophagy [56]. Moreover, EVs derived from OS cells contribute to disease progression by modulating cancer cell autophagy, drug resistance, immune responses, angiogenesis, and metastasis [57–60]. These findings highlight the potential of EVs as promising therapeutic targets for inhibiting these tumor-promoting processes in OS.

In the present work, tumors with low expression of both ATG4B and VEGF were associated with a good histological response to chemotherapy, indicating that dual inhibition of these pathways may enhance therapeutic efficacy and might be a predictor of a good response to neoadjuvant chemotherapy.

The limitations of this study include its retrospective design and relatively small sample size. Future studies should validate these findings in larger cohorts and explore the molecular mechanisms underlying the ATG4B-VEGF axis in OS. Additionally, preclinical studies are needed to evaluate the efficacy of combined ATG4B and VEGF inhibition in OS models.

## Conclusions

In our study, several key findings were observed. First, ATG4B expression was significantly upregulated in OS patients. Second, high levels of ATG4B were associated with poor prognosis and chemotherapy resistance in OS patients. Third, VEGF was found to be overexpressed in OS, and this overexpression was significantly associated with adverse prognostic factors. Fourth, a positive correlation was detected between ATG4B and VEGF expression in tumor cells, and a lack of both markers could be a predictor of a good response to neoadjuvant chemotherapy.

## Data Availability

No datasets were generated or analysed during the current study.
